# *Campylobacter coli* in Organic and Conventional Pig Production in France and Sweden: Prevalence and Antimicrobial Resistance

**DOI:** 10.3389/fmicb.2017.00955

**Published:** 2017-05-29

**Authors:** Isabelle Kempf, Annaelle Kerouanton, Stéphanie Bougeard, Bérengère Nagard, Valérie Rose, Gwénaëlle Mourand, Julia Osterberg, Martine Denis, Björn O. Bengtsson

**Affiliations:** ^1^French Agency for Food, Environmental and Occupational Health and Safety (Anses), Mycoplasmology – Bacteriology Unit, Bretagne Loire UniversityPloufragan, France; ^2^French Agency for Food, Environmental and Occupational Health and Safety (Anses), Hygiene and Quality of Poultry and Pig Products Unit, Bretagne Loire UniversityPloufragan, France; ^3^French Agency for Food, Environmental and Occupational Health and Safety (Anses), Swine Epidemiology and Welfare Research Unit, Bretagne Loire UniversityPloufragan, France; ^4^Department of Animal Health and Antimicrobial Strategies, National Veterinary InstituteUppsala, Sweden

**Keywords:** *Campylobacter*, pig, organic production, conventional production, antimicrobial resistance

## Abstract

The purpose of the study was to evaluate and compare the prevalence and antimicrobial resistance of *Campylobacter coli* in conventional and organic pigs from France and Sweden. Fecal or colon samples were collected at farms or at slaughterhouses and cultured for *Campylobacter*. The minimum inhibitory concentrations of ciprofloxacin, nalidixic acid, streptomycin, tetracycline, erythromycin, and gentamicin were determined by microdilution for a total of 263 French strains from 114 pigs from 50 different farms and 82 Swedish strains from 144 pigs from 54 different farms. Erythromycin resistant isolates were examined for presence of the emerging rRNA methylase *erm*(B) gene. The study showed that within the colon samples obtained in each country there was no significant difference in prevalence of *Campylobacter* between pigs in organic and conventional productions [France: conventional: 43/58 (74%); organic: 43/56 (77%) and Sweden: conventional: 24/36 (67%); organic: 20/36 (56%)]. In France, but not in Sweden, significant differences of percentages of resistant isolates were associated with production type (tetracycline, erythromycin) and the number of resistances was significantly higher for isolates from conventional pigs. In Sweden, the number of resistances of fecal isolates was significantly higher compared to colon isolates. The *erm*(B) gene was not detected in the 87 erythromycin resistant strains tested.

## Introduction

The organic pig sector is still a minority production in Europe, accounting in 2010 for approximately 0.33% (livestock units) in the EU-27. Organic production reached 0.9 million heads in 2011 and 0.6% of the total pig production of the EU-15 (European-Commission, 2013). France was the third largest producer (165,518 heads) behind Germany and Denmark (European-Commission, 2013). Previous studies have shown that the European organic production systems may differ considerably between countries with regards to housing, management, animal health, and welfare issues (Dippel et al., 2014; Edwards et al., 2014; Früh et al., 2014; Prunier et al., 2014a,b). However, the EU regulations for organic farming (Council Directives 2007/834/EC and 2008/889/EC) require that animals have outdoor access. Moreover, the preventive use of chemically synthesized allopathic medicinal products is not permitted. For treatment of sick animals, chemically synthesized allopathic veterinary medicinal products including antibiotics, may be used when necessary. However, this is only allowed when the use of phytotherapeutic, homeopathic, and other products is inappropriate^1^. In addition, if an animal or a group of animals receive more than one course of treatment and if their productive lifecycle is less than 1 year, the livestock concerned, or produce derived from them, may not be sold as organic products.

It may be possible that organic pigs may receive fewer antibiotics than conventionally raised animals and therefore harbor antimicrobial resistant bacteria less frequently (Young et al., 2009). This could indeed represent an interesting advantage as antimicrobial resistance is recognized as a major hazard for farmers, technicians, or veterinarians potentially exposed by direct or indirect contact with animals (Alali et al., 2010; Huijbers et al., 2015), or for consumers exposed via the food chain (Verraes et al., 2013). Thus, two aims of the SafeOrganic project (restrictive use of antibiotics in organic animal farming – a potential for safer, high quality products with less antibiotic resistant bacteria^2^) were to compare antimicrobial resistance of intestinal *Escherichia coli* (Osterberg et al., 2016) and of intestinal *Campylobacter coli* from conventional and organic pigs.

Campylobacteriosis in humans was the most commonly reported zoonosis in the European Union in 2015 (EFSA, 2016). When the species information was provided, *Campylobacter jejuni* accounted for 81.0% and *C. coli* for 8.4% of the human cases although in France the *C. coli* percentage was 15.25% (Sifre et al., 2015). Poultry and pigs are recognized as reservoirs of *Campylobacter* (Quintana-Hayashi and Thakur, 2012) and *C. coli* is the main species in pigs (Avrain et al., 2004). When treatment of human campylobacteriosis is needed, quinolones, macrolides and tetracyclines are the drugs of choice (Sifre et al., 2015).

In this paper we present the results from the SafeOrganic project investigating *C. coli* from pigs sampled in two different countries (Sweden and France) and evaluate differences possibly linked to production type and country. In addition, as macrolide-resistant *C. coli* were detected, the presence of the macrolide-resistance mechanism encoded by the rRNA methylase *erm*(B) gene, which has recently been identified in *Campylobacter* isolates (Zhang et al., 2016), was investigated.

## Materials and Methods

### Sampling

In France, colon contents of pigs from 31 organic and 31 conventional batches (group of pigs from the same farm, sampled on the same day at slaughterhouse or at farm) were collected in one slaughterhouse during April to October 2012. From each batch, as far as possible, two pigs were sampled, in total 58 conventional and 56 organic ones. As some French farms sent pigs to slaughterhouse on different dates, the samples originated from 29 and 21 unique conventional and organic farms respectively.

In Sweden, colon content of approximately 6 months old pigs from 18 organic and 18 conventional batches were collected at four different slaughterhouses from August 2012 to October 2013. From each batch two pigs were sampled, in total 72 different pigs. In addition, from each organic farm and from 18 conventional farms (not the same as sampled at slaughter), fresh feces were collected on farm from two pigs within 1 week before slaughter, in total 72 different pigs. Sampling feces from live animals without any manipulation of the animals requires no ethical permission according to Swedish legislation (SJVFS 2015:24). As the organic farms were sampled both at farm and at slaughterhouses the samples originate from 18 and 36 unique organic and conventional farms, respectively.

### *Campylobacter* Collection

In France, colon contents were diluted 10-fold in tryptone salt and streaked on Karmali plates (Oxoid, Thermo Fisher Scientific, France). Plates were incubated at 37°C in a microaerophilic atmosphere (5% O_2_, 10% CO_2_, 85% N_2_) (Air Liquide, France) for 48 h. Presence of colonies with small curved bacilli and spiraling “corkscrew” motility under phase contrast microscopy was checked on Karmali plates and isolates were then sub-cultured on blood agar plates for 24 h at 37°C for *Campylobacter* confirmation, as described in the ISO 10272 method (ISO, 2006), and for species identification. A few colonies from the bacterial culture were suspended in 200 μL of TE buffer (10 mmol L^-1^ Tris-HCl, 1 mmol L^-1^ EDTA, pH 7.6) for species identification by PCR. DNA was extracted by placing the samples at 95°C for 10 min. After low centrifugation (5,000 *g* for 2 min), 10 μL of the supernatant was diluted in 90 μL TE buffer. Multiplex-PCR was used to confirm the genus of the bacterial isolates and to identify them at the species level as described by Wang et al. (2002). This multiplex-PCR was used in our study for identification of the following five *Campylobacter* species: *C. jejuni, C. coli, C. lari, C. fetus*, and *C. upsaliensis*. Five μL of DNA were used for amplification. PCR products were visualized by the electrophoresis of 10 μL aliquots of each amplification product, for 3 h at 100 V, in a 2% agarose gel stained with GelRed (Thermo Fisher Scientific). As far as possible, up to four isolates per pig were stored at -80°C pending further analysis, resulting in a maximum of eight isolates per sampled batch.

In Sweden, one loop (10 μL) of colon contents or feces was spread on Preston selective agar (SVA, Sweden) and cultured at 42°C for 48 h in a microaerophilic atmosphere (6–13% O_2_, 3–10% CO_2_, 85% N_2_) generated using the Campygen system (Oxoid, United Kingdom). Colonies on Preston plates with a morphology suggesting *Campylobacter* were examined for presence of small curved bacilli and spiraling “corkscrew” motility under phase contrast microscopy and for production of oxidase and catalase by standard procedures. Colonies meeting the morphologic criteria on microscopy and producing oxidase and catalase were sub-cultured on blood agar plates for 24 h at 37°C. Before antimicrobial susceptibility testing, all isolates were identified to species by the MALDI Biotyper system (Bruker Daltonics, Bremen, Germany). Mass spectra were compared against 4613 spectra in the MALDI Biotyper database using the MALDI Biotyper 3.0 real-time classification software (Bruker Daltonics). One isolate of *C. coli* per pig was stored at -80°C pending further analysis.

### Antimicrobial Susceptibility Testing

Minimal inhibitory concentrations (MICs) of antimicrobials were determined using broth microdilution according to Clinical and Laboratory Standards Institute (CLSI) document M31-A3 with Sensititre^®^ plates (Biocentric, Bandol, France) in France or M45-A2 with VetMIC panels (SVA, Uppsala, Sweden) in Sweden.

In France, after a 24-h culture on blood Mueller Hinton plates, a few colonies were suspended in saline to obtain an optical density equivalent to a 0.5 McFarland standard. The suspension was diluted in blood Mueller Hinton broth to obtain approximately 5 × 10^5^ CFU/mL and distributed in a custom-made Sensititre^®^ plate. Plates were read after incubation at 41.5°C for 24 h in a microaerophilic atmosphere.

In Sweden, after a 48-h culture on blood agar, one loop (1 μL) of colony material was suspended in 2 mL saline to a density of ≈10^8^ CFU/mL. Subsequently, 50 μL was added to 10 mL of Mueller Hinton broth with 3% lysed horse blood and from this suspension VetMIC panels were inoculated with 100 μL in each well. Plates were read after incubation at 37°C for 48 h in a microaerophilic atmosphere. In both countries, *C. jejuni* ATCC 33560 was used for quality control.

Antimicrobials tested were gentamicin, streptomycin, ciprofloxacin, nalidixic acid, tetracycline, and erythromycin. MICs were interpreted according to epidemiological cut-off values issued by EUCAST^3^ as recommended by the EURL-AMR^4^. Epidemiological cut-offs for *C. coli* were >2 μg/mL for gentamicin, >4 μg/mL for streptomycin, >0.5 μg/mL for ciprofloxacin, >16 μg/mL for nalidixic acid, >2 μg/mL for tetracycline, and >8 μg/mL for erythromycin. Isolates with a non-wild-type phenotype, i.e., with a MIC above the epidemiological cutoff, were considered microbiologically resistant, for brevity hereafter referred to as resistant. The number of resistances of each isolate was calculated taking into account resistance to gentamicin, streptomycin, ciprofloxacin, tetracycline, and erythromycin.

### Detection of the *erm*(B) Gene

For erythromycin resistant strains, the presence of the *erm*(B) gene was determined by the SYBR Green PCR method. The primers ermB-F amplify a 364 bp product (**Table 1**). The test was performed in a volume of 25 μL containing 5 μL of DNA, primers (200 nM) (Huerta et al., 2013) and reaction buffer (iQ^TM^ SYBR^®^ Green, Bio-Rad, Marnes-la-Coquette, France). The qPCR detection limit of the *erm*(B) gene was 50 copies (Le Devendec et al., 2016). The *Enterococcus faecalis* JH2-2:Tn1545 *erm*(B) strain obtained from the EURL-AMR was used as a positive control.

**Table 1:** Primer sequences and the expected size of the amplicons.

Primer	Size (in bp)	Sequence (5′–3′)	Target gene	Reference
CJF	323	ACTTCTTTATTGCTTGCTGC	*C. jejuni hipO*	Wang et al., 2002
CJR		GCCACAACAAGTAAAGAAGC		
CCF	126	GTAAAACCAAAGCTTATCGTG	*C. coli glyA*	Wang et al., 2002
CCR		TCCAGCAATGTGTGCAATG		
CLF	251	TAGAGAGATAGCAAAAGAGA	*C. lari glyA*	Wang et al., 2002
CLR		TACACATAATAATCCCACCC		
CUF	204	AATTGAAACTCTTGCTATCC	*C. upsaliensis glyA*	Wang et al., 2002
CUR		TCATACATTTTACCCGAGCT		
CFF	435	GCAAATATAAATGTAAGCGGAGAG	*C. fetus sapB2*	Wang et al., 2002
CFR		TGCAGCGGCCCCACCTAT		
ermB-F	364	GATACCGTTTACGAAATTGG	*ermB*	Huerta et al., 2013
ermB-R		GAATCGAGACTTGAGTGTGC		

### Statistical Analysis

As the protocols for sampling, transport of samples, and laboratory methods were different in France and in Sweden no comparison between countries was made. However, differences in *Campylobacter* isolation rate between different matrices (colon contents and feces), and production types within each country were evaluated using the chi-square test or Fisher’s exact test. Logistic regressions were performed to evaluate the impact of the production within France and Sweden, and of the matrix type and their second-order interactions (for Sweden only) on each of the resistances under study. As up to 16 isolates per farm were analyzed, farm is considered as a random effect in the logistic model. The analyses were fitted using the geeglm() function of the geepack package in the R software^5^ (R version 3.3.1).

Multinomial logistic regression was performed to evaluate the impact of the production (for France and Sweden), and of the matrix type and their second-order interactions (for Sweden only) on the number of resistances (considered as an ordinal response). The analyses were fitted using the polr() function of the MASS package and the vglm() functions of the VGAM package, respectively, to perform the analysis and to check the proportional odds assumption.

For all tests, values of *p* < 0.05 were considered statistically significant differences.

## Results

### Isolation Rate

Isolation rates of *Campylobacter* in samples collected from pigs in organic and conventional production were not significantly different: 77% (43/56) of the organic pigs and 74% (43/58) of the conventional pigs carried the bacteria in their colon content (Chi2, *p* > 0.05). At the batch level, 27 out of 31 (87%) organic and 28 out of 31 (90%) conventional batches were positive (Fisher exact test, *p* = 1) and at the farm level, 19 out of 21 (90%) and 24 out of 29 (83%) were positive (Fisher exact test, *p* = 0.68). A total of 263 isolates were obtained from positive samples, 140 from organic and 123 from conventional pigs. All were identified as *C. coli*.

In Sweden, there was also no statistically significant difference in isolation rates between samples obtained from organic and conventional pigs. In fecal samples, the isolation rate was 61% (22/36) in organic pigs and 44% (16/36) in conventional pigs (Chi2, *p* > 0.05). In samples of colon content, the isolation rate was 56% (20/36) in organic pigs and 67% (24/36) in conventional pigs (Chi2, *p* > 0.05). There was no statistically significant difference in isolation rate between samples of feces or colon content, 53% (38/72) and 61% (44/72), respectively (Chi2, *p* > 0.05). Twelve out of 18 organic farms (67%) yielded *Campylobacter*-positive fecal samples and 12 out of 18 organic farms (67%) yielded positive colon samples; 16 out of 18 (89%) organic farms had at least one positive sample. For fecal samples collected from conventional pigs, isolates were obtained from 12 out of 18 farms (67%) and for colon samples, 16 farms out of 18 (89%) were positive. All 18 conventional farms (100%) had at least one *Campylobacter*-positive sample. All 82 isolates were identified as *C. coli*.

### Antimicrobial Resistance

The percentages of antimicrobial-resistant *C. coli* isolates are shown in **Table 2**. Resistance to erythromycin was not detected in the isolates from Sweden, but in France 62 (50%) and 25 (18%) of the isolates from conventional and organic pigs, respectively, were resistant to this antimicrobial. However, the *erm*(B) gene was not detected among these 87 erythromycin resistant isolates. Similarly, resistance to tetracycline was rare in Sweden, and was only detected in isolates from conventional pigs, with one isolate (4%) from colon content and one isolate (6%) from feces being tetracycline resistant. In contrast, resistance to tetracycline was common in France, present in 108 (77%) and 114 (93%) isolates from organic and conventional pigs, respectively. Resistance to streptomycin was frequently observed in both countries, with more than 70% of the isolates from both conventional (88 resistant isolates out of 123) and organic (104 resistant isolates out of 140) pigs in France being resistant to this antimicrobial. In Sweden, 10 out of 20 (50%) and 12 out of 24 (50%) of the isolates from colon content of both conventional and organic pigs were resistant to streptomycin and 7 out of 16 (44%) and 17 out of 22 (77%) of the isolates from feces of conventional and organic pigs, respectively. Resistance to quinolones and fluoroquinolones was common, 20–50%, in both conventional and organic pigs in France as well as in Sweden. The levels were lowest (20–30%) for isolates from French organic pigs and for isolates from colon contents of Swedish pigs of both production types. Resistance to gentamicin was not detected in Sweden and in only one of 123 isolates (1%) from conventional pigs in France. Resistance to chloramphenicol was not tested in Sweden and was not detected in the French isolates (data not shown).

**Table 2:** Number and percentages of resistance of *Campylobacter coli* isolates from samples of feces and colon content from conventional and organic pigs.

Country	Sample type	Number of isolates	Tetracycline	Erythromycin	Nalidixic acid	Ciprofloxacin	Streptomycin	Gentamicin
France	Colon Conv.	123	114 (93%)	62 (50%)	53 (43%)	53 (43%)	88 (72%)	1 (1%)
	Colon Org.	140	108 (77%)	25 (18%)	41 (29%)	39 (28%)	104 (74%)	0 (0%)
Sweden	Colon Conv.	24	1 (4%)	0 (0%)	7 (29%)	7 (29%)	12 (50%)	0 (0%)
	Colon Org.	20	0 (0%)	0 (0%)	4 (20%)	4 (20%)	10 (50%)	0 (0%)
	Fecal Conv.	16	1 (6%)	0 (0%)	8 (50%)	8 (50%)	7 (44%)	0 (0%)
	Fecal Org.	22	0 (0%)	0 (0%)	9 (41%)	9 (41%)	17 (77%)	0 (0%)

*Conv., conventional pig; Org., organic pig; ND, not determined.*

In France, there was a significant impact of the production type on resistance only for tetracycline (logistic regression, *p* = 0.007) and erythromycin (logistic regression, *p* = 0.005) with resistance to these antimicrobials being more common in isolates from conventional pigs. For Sweden, no significant associations related to production system were observed for the different antimicrobials, but there was a tendency for isolates from fecal samples to be more often resistant to quinolones and fluoroquinolones (logistic regression, *p* = 0.06) compared to isolates from colon.

For French isolates, the number of resistances of individual isolates was significantly associated with the production type (multinomial logistic regression, *p* = 1.8e-07) with higher number of resistances in isolates from conventional pigs. For Swedish isolates, the number of resistances of fecal isolates was significantly higher compared to the colon isolates (multinomial logistic regression, *p* = 0.04).

In France, when several isolates from the same pig were studied, various resistance profiles were found: thus for pigs for which four isolates were analyzed, the mean number of resistance profiles was 1.8. Similarly, when several isolates from the same farm were analyzed, several resistance profiles were usually observed: for example, when four or more isolates from a French farm were studied, 2–6 and 1–5 different resistance profiles were detected for organic and conventional farms, respectively. Likewise, when three or four isolates were obtained from a Swedish farm, a minimum of two different profiles were detected. In France, the most frequent resistance profiles were streptomycin–tetracycline (27/123, 22%), streptomycin-ciprofloxacin-tetracycline-erythromycin (19/123, 15%), streptomycin-ciprofloxacin-tetracycline (18/123, 15%) and streptomycin-tetracycline-erythromycin (17/123, 14%) for conventional production and streptomycin–tetracycline (46/140, 33%), streptomycin-ciprofloxacin-tetracycline (22/140, 16%) and tetracycline (17/140, 12%) in organic production (**Table 3**). Multidrug-resistance (resistance to three or more antimicrobial families) was more frequently observed for isolates from conventional pigs (68/123, 55% of the isolates) than for isolates from organic pigs (37/140, 26%) (Chi2, *p <* 0.001). In Sweden, the two most frequent profiles were wild-type (13/40, 32.5%) and streptomycin (12/40, 30%) for conventional production and streptomycin (17/42, 40.5%) and wild-type (12/42, 29%) for organic production. No isolate from Sweden was multidrug-resistant.

**Table 3:** Antimicrobial resistance profiles.

	France			Sweden					
	Conventional	Organic	Total	Conventional			Organic			
Resistance profile	Feces			Feces	Colon	Total	Feces	Colon	Total	Total
C				4	2	6	1	2	3	9
CT	2	2	4	1	1	2				2
CTE	13	2	15							
E	1	1	2							
GCT	1		1							
S	4	15	19	4	8	12	9	8	17	29
SC		5	5	3	4	7	8	2	10	17
SCT	18	22	40							
SCTE	19	8	27							
SE	3	3	6							
ST	27	46	73							
STE	17	5	22							
T	8	17	25							
TE	9	6	15							
W	1	8	9	4	9	13	4	8	12	25
Total	123	140	263	16	24	40	22	20	42	82

*S, streptomycin; C, ciprofloxacin; T, tetracycline; E, erythromycin; W, wild-type.*

## Discussion

### Occurrence of *C. coli*

*Campylobacter coli* was the main species isolated and the prevalence in colon contents was over 50% in both countries and in both production types. These results are in accordance with the metagenomic data for a subset of our samples reported from another study within the SafeOrganic project. Gerzova et al. (2015) found the 16S rRNA gene of the genus *Campylobacter* in all six French samples of pig colon contents investigated and in three out of five individual samples of colon contents and one pooled sample from Sweden. Such a high prevalence of *Campylobacter* in pigs is frequently observed in many countries such as France (Avrain et al., 2004), Great Britain (Milnes et al., 2008), the United States (Tadesse et al., 2011), or Canada (Huang et al., 2015) but slightly lower prevalences have sometimes been reported, as in Japan (Haruna et al., 2013) and in Sweden (Bywater et al., 2004). Levels from 0 to 92.7% were reported for European member and non-member states (EFSA and ECDC, 2015). These differences in prevalence of *Campylobacter* in pigs are probably related to true differences between countries but also to differences in the protocols used in the different studies, such as farm or slaughterhouse sampling, type and number of samples, individual or pooled samples, enrichment or direct plating and number and type of media. However, these data clearly indicate that maintaining pig herds free of *Campylobacter* is likely to be challenging. Nevertheless, *Campylobacter*-free pig herds were described in Norway, although these herds were located in remote areas far away from conventional pig herds (Kolstoe et al., 2015).

In the current study there was no difference in the prevalence of *C. coli* in organic and conventional production, neither in France nor in Sweden. This is consistent with the metagenomic study of Gerzova et al. (2015) on the samples from this study where there was no significant difference in the composition of the microbiota of organic and conventional pigs. In the study reported by Rollo et al. (2010), concerning 60 conventional farms and 35 antibiotic-free farms in Midwestern United States, *Campylobacter* was detected in 35.8 and 36.4% of pigs, respectively, with no significant difference between these two production types. A similar observation was made by Gebreyes et al. (2005), with prevalences of 55 and 56.3% for extensive and intensive pig rearing systems, respectively, and by Quintana-Hayashi and Thakur (2012), who monitored the presence of *Campylobacter* in the environment of conventional and antimicrobial-free pig production systems and found no difference. Likewise, in other studies no significant differences could be detected for prevalence of *Campylobacter* in conventional and organic dairy productions (Sato et al., 2004), in meat from free-range and conventional broilers (Economou et al., 2015), in organic, antibiotic-free and conventional retail chicken breasts (Mollenkopf et al., 2014) and in conventional and organic layer hens (Schwaiger et al., 2008). However, *C. jejuni* was significantly less frequently found in organically raised than in conventionally raised layer hens (Kassem et al., 2017). Inversely the meta-analysis of Young et al. (2009) showed that at slaughter, *Campylobacter* was more common in organic than in conventional broilers. Various factors such as access to outdoor runs, animal density, age at slaughter or slaughterhouse processing conditions may partially explain these differences.

### Antimicrobial Resistance

The resistance ratios registered in this study for conventional pigs can be compared with data reported for *C. coli* from pigs by France in the 2011 mandatory monitoring of production animals in the EU (EFSA-ECDC, 2013) and for Sweden in the 2011 and 2015 monitorings (EFSA-ECDC, 2013, 2017). In the mandatory monitoring scheme, the herds sampled are representative of the national production, which means that, for both France and Sweden, the samples originate almost exclusively from conventional herds. The data on resistance presented in this study were similar to the results of the EFSA and ECDC monitorings, although data on streptomycin and multidrug resistance were not included in the latter.

Several studies have investigated the occurrence of antimicrobial resistance in organic or antibiotic-free production systems in comparison to conventional systems. Although several factors may differ between conventional and organic productions, such as animal density, access to outdoor areas, and age at slaughter, the most important factor for selection and persistence of antimicrobial resistance is probably use of antimicrobials as recognized in other situations (WHO, 2012). To investigate possible differences in antimicrobial resistance between production types, the susceptibility of commensal *Enterobacteriaceae* or *E. coli* is sometimes the chosen criteria as these bacteria are present in most fecal samples, are easy to isolate and analyze, and are considered a major resistance gene reservoir. Thus in the course of the SafeOrganic project, Osterberg et al. (2016) showed that, in Denmark, Italy, France and Sweden, resistance to ampicillin, streptomycin, sulphonamides, and trimethoprim was significantly less frequent in *E. coli* strains from organic than from conventional pigs. This was also observed in France for chloramphenicol. Several antimicrobials were tested for both the *E. coli* and the *C. coli* isolates collected during this project (ciprofloxacin, gentamicin, nalidixic acid, streptomycin, and tetracycline). The lower ratio of resistance to tetracycline reported for *E. coli* from organic compared to conventional pigs in France, but not in Sweden, was also observed for *Campylobacter* in France. For quinolones and fluoroquinolones, no differences between production types were registered for either *Campylobacter* or *E. coli* in both countries. Streptomycin resistance was significantly different in the two production types in both countries for *E. coli* but not for *C. coli*. Thus, within a given country, the result of a comparison of the antimicrobial susceptibility between two animal production types will depend on the bacterial species tested, which may differ in their evolution and the persistence of their antimicrobial resistance. Such differences in the susceptibility of bacterial species are probably related to various factors including the biological cost of resistance, which will influence the rate at which a bacterial species persists or regains wild-type status after reduced use of a specific antimicrobial. It was previously demonstrated that for example fluoroquinolones, not always impose a biological cost in *Campylobacter* (Luo et al., 2005; Zeitouni and Kempf, 2011). This phenomenon could explain the rather high levels of quinolone and fluoroquinolone resistance in *Campylobacter* strains from organic pigs observed in both France and Sweden. Indeed, in the United States, Zawack et al. (2016) showed that the withdrawal of enrofloxacin for use in poultry 10 years ago had no effect on the occurrence of ciprofloxacin resistance in *Campylobacter* from chickens, and El-Adawy et al. (2015) reported surprisingly high levels of resistance to ciprofloxacin in *Campylobacter* isolates from organically raised turkeys in Germany, which they attributed to the persistence of strains during the transition from conventional to organic production. High levels of fluoroquinolone resistance was also detected in *Campylobacter* isolates from chicken in organic productions in Portugal (Fraqueza et al., 2014).

The persistence of resistance can also be linked to co-selection whereby the use of one antimicrobial selects for resistance also to other antimicrobials. In Japan Ozawa et al. (2012) used logistic regression to demonstrate that the use of tetracyclines within the 6 months prior to the survey was associated with chloramphenicol resistance of *C. coli* in pigs. This co-selection was explained by the fact that all chloramphenicol-resistant isolates were also resistant to tetracycline.

Besides the analysis of various indicator or zoonotic bacteria (Young et al., 2009), another criteria that can be analyzed is the abundance of antimicrobial resistance genes. However, in the SafeOrganic project, the abundance of a few selected genes [*strA, sul1, sul2, tet*(A), and *tet*(B)] was found to be linked to the country but not to the production type (Gerzova et al., 2015). This rather unexpected result may be due to a lower discriminatory power of the qPCR method compared to culturing. Moreover, the resistance genes that were analyzed may be harbored by many different bacterial species, e.g., *tet*(A) can be found in *Enterobacteriaceae, Aeromonadaceae, Pseudomonadaceae, Vibrionaceae* and others, and each bacteria may contain different genes encoding for the same antimicrobial family. For example, *Escherichia* can contain *tet*(A), *tet*(B) *tet*(C), *tet*(D), *tet*(E), *tet*(I), and others (Chopra and Roberts, 2001), which may explain why for a given antimicrobial, results may be different in terms of the susceptibility ratios of *E. coli* or *Campylobacter*, or the abundance of resistance genes.

Our results show associations between antimicrobial resistance of *C. coli* in France and type of production, in particular for tetracycline and macrolides. Different studies have previously focused on the link between antimicrobial consumption and resistance of *C. coli* in pigs. In Japan, Ozawa et al. (2012) found significant associations, with odds ratios around 10, between the use of macrolides and phenicols and resistance to erythromycin and chloramphenicol, respectively. In the United States, the *C. coli* strains from antibiotic-free pigs were less often resistant to tetracycline and to clindamycin than those from conventional pigs (Quintana-Hayashi and Thakur, 2012) and the most common profile (resistance to ciprofloxacin, nalidixic acid, and tetracycline) observed in conventional pigs was found in only one antibiotic-free pig. Rollo et al. (2010) had similar findings with significant differences for resistance to macrolides (erythromycin and azithromycin) and tetracycline between farm types. They noted that resistance to these antimicrobials decreased with the number of years that a farm had been antibiotic-free.

The results of the current study are globally consistent with the significant positive associations between consumption of macrolides in animals in different European countries and resistance in *C. coli* and *C. jejuni* in animals in these countries, as reported in the joint ECDC/EFSA/EMA report on integrated analysis of antimicrobial consumption and occurrence of antimicrobial resistance (ECDC et al., 2015). However, the authors of the report note that there are limitations to their study since it is based on consumption data for all species of food-producing animals and on only a small number of countries reporting resistance data for *Campylobacter*.

Notably, in the current study erythromycin resistance was not found in isolates from Sweden. In a study evaluating data on resistance from the 2011 EU summary report in relation to use of antimicrobials as reported to EMA, the absence of macrolide-resistance in *C. coli* from pigs in Sweden was attributed to withdrawal of the macrolide tylosin as growth promoter as early as 1986 and to the restricted therapeutic use of this antimicrobial (Garcia-Migura et al., 2014). In contrast, many *C. coli* strains from French conventional or organic pigs were resistant to erythromycin. Previous studies have shown that resistance to macrolides in French isolates of *C. coli* from pigs was associated with mutation in the 23S rRNA genes and efflux pumps (Payot et al., 2004). However, the recent emergence of macrolide-resistance linked to the transmissible *erm*(B) gene, encoding an rRNA methylase, prompted us to screen for this gene in our recent strains. None contain the *erm*(B) gene. To our knowledge, this gene has only been detected in Chinese isolates from humans, swine, chickens, and ducks and in one *C. coli* isolated from a broiler in Spain (Florez-Cuadrado et al., 2016). The absence of *erm*(B) in our strains is reassuring, but surveillance must be maintained.

## Conclusion

The protocol of this study enabled us to evaluate the prevalence and antimicrobial resistance in *C. coli* from conventional and organic pigs in Sweden and France. We could show that within each country there was no significant difference in prevalence of *C. coli* between organic and conventional production. We also showed that in France there were significant differences between production types in occurrence of resistance to tetracycline and erythromycin and the number of resistances of *C. coli* isolates, resistance being more common in conventional than in organic production. In Sweden, no differences in resistance between the two systems were detected.

## Author Contributions

IK: contributed to the design of the work, contributed to the acquisition, analysis and the interpretation of the data, wrote the paper. AK: contributed to the design of the work, participated to the field study, contributed to the acquisition, analysis and the interpretation of the data, wrote the paper. SB: contributed to the acquisition, analysis and the interpretation of the data, wrote the paper. BN: participated to the field study, contributed to the acquisition, analysis and the interpretation of the data, final approval of the submitted version. VR: participated to the field study, contributed to the acquisition, analysis and the interpretation of the data, final approval of the submitted version. GM: contributed to the acquisition, analysis and the interpretation of the data, final approval of the submitted version. JO: contributed to the design of the work, participated to the field study, contributed to the acquisition, analysis and the interpretation of the data, wrote the paper. MD: contributed to the design of the work, participated to the field study, contributed to the acquisition, analysis and the interpretation of the data, wrote the paper. BB: contributed to the design of the work, participated to the field study, contributed to the acquisition, analysis and the interpretation of the data, wrote the paper.

## Conflict of Interest Statement

The authors declare that the research was conducted in the absence of any commercial or financial relationships that could be construed as a potential conflict of interest.

## Footnotes

1 https://ec.europa.eu/agriculture/organic/eu-policy/eu-rules-on-production/livestock_en

2 http://www.coreorganic2.org/

3 www.eucast.org

4 http://www.crl-ar.eu/data/images/reports/rapport-eurl%20salm-camp%20eqas%202010.pdf

5 https://cran.r-project.org/web/packages/geepack/index.html
